# Hybrid Genotype of *Anisakis simplex* (s.s.) and *A. pegreffii* Identified in Third- and Fourth-Stage Larvae from Sympatric and Allopatric Spanish Marine Waters

**DOI:** 10.3390/ani11082458

**Published:** 2021-08-21

**Authors:** Xavier Roca-Geronès, M. Magdalena Alcover, Carla Godínez-González, Isabel Montoliu, Roser Fisa

**Affiliations:** Laboratory of Parasitology, Department of Biology, Health and Environment, Faculty of Pharmacy and Food Sciences, University of Barcelona, Av. Joan XXIII 27-31, 08028 Barcelona, Spain; xavierroca@ub.edu (X.R.-G.); mmagdalenaalcoveramengual@ub.edu (M.M.A.); carla8610@gmail.com (C.G.-G.); montoliu@ub.edu (I.M.)

**Keywords:** *Anisakis simplex* (s.s.), *Anisakis pegreffii*, hybrid genotype, in vitro culture, Spanish marine waters, genotyping approach

## Abstract

**Simple Summary:**

The nematode species *Anisakis simplex* sensu stricto (s.s.) and *Anisakis pegreffii* are wormlike parasites found in commonly consumed fish and are the main cause of human anisakiasis. Outwardly, the two nematodes are extremely similar and difficult to distinguish, especially in their larval forms. Genetic analysis has discovered the existence of a hybrid between these two “sibling species”, but its identification is a controversial matter, as results differ according to the specific region of the DNA analysed. The aim of our work was to confirm the presence of this hybrid genotype in fish off the Spanish coast and to obtain fourth-stage larvae in the laboratory to study if different genotypes are associated with different growth behaviour. Our results confirm that hybrid genotypes can be overestimated if identification is based on a particular molecular marker. We also obtained fourth-stage larvae with a hybrid genotype, which has not been reported previously. These findings are valuable for the taxonomic classification of *Anisakis* species, and for further epidemiological and biomedical research.

**Abstract:**

The sibling species *Anisakis simplex* (s.s.) and *Anisakis pegreffii* are parasites of marine mammals and fish worldwide and the main causative agents of human anisakiasis. In sympatric areas, a hybrid genotype between the two species has been identified, mainly in third-stage larvae, but rarely in fourth-stage and adult forms. The aim of this study was to confirm the presence of hybrid genotypes in larvae parasitizing fish caught in sympatric and allopatric Spanish marine waters, the North-East Atlantic and West Mediterranean, respectively, and to study possible differences in the growth behaviour between genotypes. Of the 254 molecularly analysed larvae, 18 were identified as hybrids by PCR-RFLP analysis of the rDNA ITS region, 11 of which were subsequently confirmed by EF1 α-1 nDNA gene sequencing. These results therefore indicate an overestimation of hybrid genotypes when identification is based only on the ITS region. We also report the detection of a hybrid specimen in a host from the West Mediterranean, considered an allopatric zone. Additionally, fourth-stage larvae with a hybrid genotype were obtained in vitro for the first time, and no differences were observed in their growth behaviour compared to larvae with *A. simplex* (s.s.) and *A. pegreffii* genotypes.

## 1. Introduction

Ascaridoid nematodes of the family Anisakidae include species with sanitary and/or commercial impact that are found worldwide in fish and marine mammals [1,2,3]. The life cycle of these parasites includes cetaceans and pinnipeds as definitive hosts, a wide range of fish and cephalopods as paratenic and/or intermediate hosts, and crustaceans as first intermediate hosts [2]. Species of the genus *Anisakis* are the main causative agents of human anisakiasis, an emerging disease with gastrointestinal and/or allergic symptoms acquired by eating raw or undercooked fish parasitized with larvae [4,5,6,7]. The disease is mainly caused by third-stage larvae (L3) of the sibling species *Anisakis simplex* sensu stricto (s.s.) and *Anisakis pegrefii*, which are included in the *A. simplex* sensu lato (s.l.) complex, together with *A. berlandi*, and are morphologically indistinguishable [8,9].

*A. simplex* (s.s.) and *A. pegreffii* have a worldwide distribution, with allopatric areas where only one species is found, and sympatric areas where the two overlap [2]. In sympatric areas, such as the North-East Atlantic and the North-West Pacific, both species can also share hosts and commonly co-infect the same definitive or intermediate/paratenic host. In these areas, hybrid forms between the two sibling species have been detected [9,10,11]. However, despite the large number of L3 larvae described with a hybrid genotype, reports of hybrid fourth-stage larvae (L4) and adult specimens in definitive hosts are scarce [11].

Until recent years, hybrid forms among *A*. *simplex* (s.l.) sibling species were identified mainly by PCR-RFLP of the rDNA ITS region, although it has been suggested that this method leads to an overestimation of hybrids [12,13]. Alternative multi-marker nuclear genotyping approaches have been proposed, such as the use of allozyme markers or sequencing of the elongation factor 1 alpha 1 (EF1 α−1) nDNA [12].

The aim of the present study was to confirm the presence of hybrid genotypes between *A. simplex* (s.s.) and *A. pegreffii* in larvae parasitizing fish caught in sympatric and allopatric Spanish marine waters using a multi-marker genotyping approach. Moreover, to gain insight into the behaviour of the hybrid genotype, special emphasis was placed in obtaining further developmental stages of the two sibling species by in vitro culture.

## 2. Materials and Methods

### 2.1. Fish Samples, Larvae Collection, and Morphological Identification

Two teleostean fish species commonly consumed in Spain were analysed for the presence of *A. simplex* (s.l.) larvae: horse mackerel (*Trachurus trachurus*) (*n* = 52) and blue whiting (*Micromesistius poutassou*) (*n* = 98). All hosts were acquired dead from markets in Barcelona during 2015-17 and came from two fishing areas, the sympatric area of the North-East Atlantic Ocean, corresponding to zone 27.8 of the Food and Agriculture Organization of the United Nations (FAO), and the allopatric region of the Western Mediterranean Sea, corresponding to FAO zone 37.1.1. The fish were dissected and viscera were examined under the stereomicroscope for the detection and isolation of nematode larvae. *Anisakis* larvae were preserved in 70% ethanol and studied microscopically. Larval specimens were cut in three portions: the anterior and posterior parts were mounted in lactophenol for morphological identification, following the criteria of Berland [14], and the central portion was used for molecular analysis.

### 2.2. In Vitro Culture of Anisakis Larvae

A selection of L3 larvae of *A. simplex* (s.l.) (*n* = 200), isolated from North-East Atlantic blue whiting, were cultured in vitro to obtain further developmental stages. Larvae were first placed in antibiotic and antimycotic solution for axenization, as reported by Iglesias et al. (1997), [15] and subsequently cultured in commercial medium RPMI-1640 + 20% (*v*/*v*) FBS (foetal bovine serum) + 0.1% *w/v* pepsin (1:10,000 NF), adjusting the pH to 4.0, at 37 °C with an atmosphere of 5% CO_2_ [16]. Culture plates were observed daily under the inverted microscope to monitor larval evolution. Once moulted into L4 larvae, a subsample of 90 specimens was fixed and preserved in 70% ethanol for morphological and molecular identification. The remaining larvae continued to be cultured in vitro to obtain adult forms.

### 2.3. Molecular Identification

Genomic DNA was isolated using the Pure PCR Template Extraction Kit (Roche), according to the manufacturer’s protocol. Specific identification of larvae was carried out by PCR-RFLP analysis of the complete rDNA ITS region using NC2 and NC5 primers with a hybridization temperature of 55 °C. DNA amplification products were digested with restriction endonucleases *Hinf*I and *Hha*I at 37 °C for 90 min. The digested products were subjected to electrophoresis in 2% agarose gel and visualized by a UV light Illuminator [17]. To confirm the detected hybrid genotypes, the elongation factor 1 alpha 1 (EF1 α-1) of the nDNA region of these specimens, as well as of *A. simplex* (s.s.) and *A. pegreffii* larvae identified by PCR-RFLP, was amplified and sequenced from the isolated DNA using EF-F and EF-F primers with a hybridization temperature of 58 °C [12].

### 2.4. Statistical Analysis

The nonparametric chi-squared test (χ2) was used to assess differences in the molecular identification methodology, the PCR-RFLP and the EF1 α-1 sequencing. The statistical analysis was performed using SPSS v22 software and the level of significance was set to *p* < 0.05.

## 3. Results

### 3.1. Morphological Identification and In Vitro Culture of A. simplex (s.l.)

All larvae obtained from the dissected fish were morphologically identified as L3 larvae of *A. simplex* (s.l.). After four days, 100% of the L3 larval specimens selected for in vitro culture moulted to L4 larvae, characterized by the loss of the cephalic tooth, the presence of developed lips, a marked striated cuticle, the absence of a mucron, and signs of moult in the culture media. The motility of L4 larvae declined progressively until their death, which occurred between days 7 and 55 of culture (on average, day 34), and adult specimens were not obtained.

### 3.2. Molecular Identification

**Figure 1:**
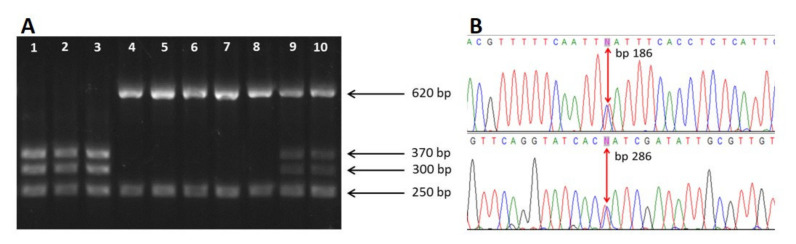
(**A**) Restriction fragment length polymorphism patterns of the ITS region of the rDNA of *Anisakis* larvae using the *Hinf*I restriction enzyme. Lines 1–3: *A. pegreffii* (370, 300, 250 bp); Lines 4–8: *A. simplex* (s.s.) (620, 250, 80 bp); Lines 9–10: Hybrid genotype between *A. pegreffIi* and *A. simplex* (s.s.) (620, 370, 300, 250, 80 bp). (**B**) Partial EF1 α-1 nDNA sequences of hybrid specimens, highlighting the two diagnostic positions, bp 186 and 286, respectively, using Sequence Scanner Software v 2.0.

A total of 254 *A. simplex* (s.l.) larvae, including L3 and L4 larvae, were molecularly identified as *A. simplex* (s.s.) (*n* = 140), *A. pegreffii* (*n* = 96) and hybrid forms (*n* = 18) according to genotype-specific patterns revealed by PCR-RFLP analysis of the rDNA ITS region (Figure 1A). The host and geographical origin of the identified specimens are shown in Table 1.

Sequencing of the EF1 α-1 nDNA was performed in all hybrid genotypes and in a representative subsample of *A. simplex* (s.s.) (*n* = 5) and *A. pegreffii* (*n* = 5) L3 larvae previously identified by PCR-RFLP. EF1 α-1 sequencing confirmed the specific identification of *A. simplex* (s.s.) and *A. pegreffii* through the observation of the corresponding nucleotides at two diagnostic positions: base pairs 186 and 286. Hybrid genotypes were confirmed in 11 specimens by the observation of a heterozygote pattern at both diagnostic positions (Figure 1B), while 7 specimens presenting the heterozygote pattern by PCR-RFLP were finally identified as *A. simplex* (s.s.) (*n* = 3) and/or *A. pegreffii* (*n* = 4) (Table 1). The chi-squared test showed significant differences when comparing the hybrid genotype identification by PCR-RFLP with their identification by the multi-marker approach (*p* < 0.01). Among the confirmed hybrid specimens, four corresponded to L3 and seven to L4 larvae. Sequences of the studied larvae were deposited in GenBank under the accession numbers MZ517160-2.

Using the multi-marker approach, *A. simplex* (s.s.) specimens were only identified in North-East Atlantic hosts, whereas *A. pegreffii* and hybrid genotypes were detected in fish from both Atlantic and Mediterranean areas. In the allopatric area of the West Mediterranean, one sample was identified with the hybrid genotype (1/44), while in the sympatric area of the North-East Atlantic, hybrid genotypes represented 4.8% of the total analysed larvae (10/210).

## 4. Discussion

The extensively used rDNA ITS region/gene is an efficient tool for the specific identification of nematodes [18,19]. However, it has been suggested that molecular techniques based only on this region have limited power regarding the identification of hybrid genotypes between *A. simplex* (s.s.) and *A. pegreffii*, and may lead to an overestimation of hybrid specimens [12,13]. This limitation is not exclusive to *Anisakis* hybrid genotypes, and the risk of using a single genetic marker has been highlighted for the specific identification of other parasites [20].

For the species included in the *A. simplex* (s.l.) complex, the two polymorphisms of the ITS region, previously designated as species-specific, are not fixed diagnostic markers, so it is not clear whether the polymorphism shared by the two taxa is caused by incomplete lineage sorting, historical introgression, or current hybridization [12]. Therefore, to elucidate the hybridization phenomena between *Anisakis* sibling species, multi-marker nuclear genotyping approaches have recently been proposed, such as the use of allozyme markers and sequencing of the EF1 α-1 nDNA [12]. The results of the present study suggest that analysis based only on the rDNA ITS region can lead to an overestimation of hybrids, with fewer being detected by EF1 α-1 nDNA gene sequencing, supported by significant statistical differences. The identification of the hybrid genotype should therefore be confirmed by the analysis of another DNA region in addition to the rDNA ITS.

The detection and/or origin of *Anisakis* hybrid genotypes remains a matter of controversy. Mladineo et al. [13], in a study using a microsatellite panel, suggested that the hybridization is caused by ancestral polymorphism, which could be a leftover of incomplete lineage sorting. However, this observation was questioned by Mattiucci et al. [8], who obtained divergent results using a different microsatellite panel. Far from disputing the utility of microsatellites as a tool for *Anisakis* sibling species identification, these authors argue that more studies are needed to clarify the levels of genetic differentiation between these species, which would open a wide field of study in *Anisakis* population genetics based on microsatellite analysis.

More recently, several authors have identified hybrids between *A. simplex* (s.s.) and *A. pegreffii* using novel diagnostic markers from the nuclear genomes, such as the beta-tubulin gene and the *nas10* nDNA region, thus expanding the range of markers available for the multi-marker genotyping approach to *Anisakis* species classification [21,22].

The detection of hybrid genotypes between *A. simplex* (s.s.) and *A. pegreffii* is of great significance for the study of speciation in the *A. simplex* (s.l.) complex. Llorens et al. [23] observed strong parent-of-origin effects in the hybrid transcript repertoire, which would be important in the evolutionary biology of *A. simplex* (s.s.) and *A. pegreffii* through gene introgression. Other authors suggest that the presence of hybrid forms between the sibling species complicates the accurate definition of genomes, transcripts, and proteins, including the assignment of genes or gene products to a determinate species [24].

Hybrid detection is also important in terms of the biomedical impact of the parasite on humans and its pathogenic potential for marine hosts. In this context, it has been suggested that *A. simplex* (s.s.), *A. pegreffii* and their hybrids have differential allergenic potential, and may induce overlapping disease responses, with a variable capacity to penetrate tissue [23,25,26]. Hybrid forms are also relevant from an epidemiological point of view, as hybridization can lead to the colonization of new hosts [27]. Moreover, the presence of hybrids reflects the dynamics of moving areas of sympatry and sheds lights on the microevolutionary processes of host–parasite ecology and definitive host migration [23].

Hybrid specimens between *A. simplex* (s.s.) and *A. pegreffii* have previously been documented in fish from sympatric areas, such as the North-East Atlantic, in agreement with their detection in two Atlantic hosts in the current study, and the Sea of Japan [10,11,28]. This phenomenon has also been observed in Spanish Mediterranean waters, especially the sympatric area of the Alboran Sea, including in horse mackerel and blue whiting, the two hosts studied here [11,29,30]. The proportion of hybrid genotypes previously reported in North-East Atlantic waters is slightly higher (around 15%) than the value we obtained.

In contrast with most reports, which indicate that hybrid genotypes are restricted to sympatric areas, in the present study a hybrid specimen was detected in the allopatric area of the West Mediterranean Sea. This could be explained by host mobility, as horse mackerel are highly migratory and could have travelled there from the Atlantic Ocean or the Alboran Sea, where *A. simplex* (s.s.) and hybrid specimens are present [31,32]. Therefore, the identification of a hybrid in the West Mediterranean highlights the possibility of finding hybrid genotypes in allopatric areas, particularly those near sympatric regions. Hybrid specimens have been previously reported in strictly allopatric areas, such as the Adriatic, the Tyrrhenian or the Aegean Sea [13,33,34], but all the studies, except Mladineo et al. [13], based the identification on the rDNA ITS region, which provides insufficient evidence for the presence of hybrids.

In the present work, a hybrid genotype was also identified in seven L4 larval specimens obtained by in vitro culture, which showed no differences in in vitro culture behaviour or the moulting process in comparison with *A. simplex* (s.s.) and *A. pegreffii*. No L4 larvae with a hybrid genotype have been described in definitive hosts and few hybrid adults have been identified [10,21,35]. Consequently, it has been suggested that *Anisakis* hybrid specimens have low fertility and/or reduced fitness, which could affect their survival and therefore their detection and identification in definitive hosts [12,36]. Nevertheless, another factor could be the difficult recovery of these developmental stages in contrast with the readily obtainable L3 larvae. It has also been proposed that hybrid forms might only be able to survive in hosts when accompanied by their parental species [23], but in our case all in vitro-cultured L3 larvae, growing individually without competition, reached the fourth developmental stage, with no specific difference between genotypes.

Although a hybrid genotype was identified in the cultured L4 larvae, reported here for the first time, no adult specimens were obtained on continuing the culture, regardless of the genotype. Despite this, in vitro culture of *Anisakis* species constitutes a useful tool in the study of developmental stages in the nematode life cycle. In several recent studies, in vitro culture was employed to analyse the transcriptome and gene expression in L4 larvae of *A. simplex* (s.s.), *A. pegreffii* and their hybrids, as well as for morphological and molecular characterization [9,37,38].

## 5. Conclusions

Our results confirm that a multi-marker genetic approach is needed to identify hybrid genotypes between *A. simplex* (s.s.) and *A. pegreffii*, as analysis based only on the ITS rDNA region may lead to an over-detection. Notably, we identified a hybrid specimen in the allopatric area of the Western Mediterranean, which was likely due to the migration of horse mackerel. Additionally, fourth-stage larval hybrids, which were obtained in vitro for the first time in this study, presented no moulting or behavioral differences with regard to *A. simplex* (s.s.) or *A. pegreffii* larvae at the same stage of development.

## Figures and Tables

**Table 1:** Distribution and molecular identification of *A. simplex* (s.l.) L3 and L4 larvae.

Developmental Stage	Area	Host Species	*A.s.* (s.s.) ^1^	*A.p.* ^1^	H g ^1^	H g ^2^
L3	West Mediterranean	*T. trachurus*	-	28	3	1
		*M. poutassou*	-	13	-	-
	North-East Atlantic	*T. trachurus*	10	11	1	1
		*M. poutassou*	74	20	5	2
L4	North-East Atlantic	*M. poutassou*	56	24	9	7
Total			140	96	18	11

*A.s.* (s.s.): *A. simplex* (s.s.); *A.p.*: *A. pegreffii*; H g: Hybrid genotype; ^1^: species identification using only PCR-RFLP of ITS rDNA; ^2^: confirmation of hybrid genotype using EF1 α-1 nDNA sequencing.

## Data Availability

The data presented in this study are available on request from the corresponding author.

## References

[1] Levsen A., Svanevik C.S., Cipriani P., Mattiucci S., Gay M., Hastie L.C., Bušelić I., Mladineo I., Karl H., Ostermeyer U. (2018). A survey of zoonotic nematodes of commercial key fish species from major European fishing grounds—Introducing the FP7 PARASITE exposure assessment study. Fish. Res..

[2] Mattiucci S., Cipriani P., Levsen A., Paoletti M., Nascetti G. (2018). Molecular epidemiology of *Anisakis* and Anisakiasis: An ecological and evolutionary road map. Adv. Parasitol..

[3] Smaldone G., Abollo E., Marrone R., Bernardi C.E.M., Chirollo C., Anastasio A., Del Hierro S.P. (2020). Risk-based scoring and genetic identification for anisakids in frozen fish products from Atlantic FAO areas. BMC Vet. Res..

[4] Audícana M.T., Ansotegui I.J., Fernández de Corres L., Kennedy M.W. (2002). *Anisakis simplex*: Dangerous dead and alive?. Trends Parasitol..

[5] Mattiucci S., D’Amelio S., Bruschi F. (2014). Helminth Infections and Their Impact on Global Public Health.

[6] Smaldone G., Ambrosio R.L., Marrone R., Ceruso M., Anastasio A. (2020). *Anisakis* spp. Larvae in deboned, in-oil fillets made of anchovies (*engraulis encrasicolus*) and sardines (*sardina pilchardus*) sold in eu retailers. Animals.

[7] Smaldone G., Marrone R., Palma G., Sarnelli P., Anastasio A. (2017). Preliminary study on the inactivation of anisakid larvae in baccalà prepared according to traditional methods. Ital. J. Food Saf..

[8] Mattiucci S., Bello E., Paoletti M., Webb S.C., Timi J.T., Levsen A., Cipriani P., Nascetti G. (2019). Novel polymorphic microsatellite loci in *Anisakis pegreffii* and *A. simplex* (s.s.) (Nematoda: Anisakidae): Implications for species recognition and population genetic analysis. Parasitology.

[9] Roca-Geronès X., Segovia M., Godínez-González C., Fisa R., Montoliu I. (2020). *Anisakis* and *Hysterothylacium* species in Mediterranean and North-East Atlantic fishes commonly consumed in Spain: Epidemiological, molecular and morphometric discriminant analysis. Int. J. Food Microbiol..

[10] Umehara A., Kawakami Y., Matsui T., Araki J., Uchida A. (2006). Molecular identification of *Anisakis simplex* sensu stricto and *Anisakis pegreffii* (Nematoda: Anisakidae) from fish and cetacean in Japanese waters. Parasitol. Int..

[11] Abattouy N., Valero A., Lozano J., Barón S.D., Romero C., Martín-Sánchez J. (2016). Population genetic analysis of *Anisakis simplex* s.l. and *Anisakis pegreffii* (Nematoda, Anisakidae) from parapatric areas and their contact zone. Parasite Epidemiol. Control.

[12] Mattiucci S., Acerra V., Paoletti M., Cipriani P., Levsen A., Webb S.C., Canestrelli D., Nascetti G. (2016). No more time to stay ‘single’ in the detection of *Anisakis pegreffii*, *A. simplex* (s.s.) and hybridization events between them: A multi-marker nuclear genotyping approach. Parasitology.

[13] Mladineo I., Bušelić I., Hrabar J., Vrbatović A., Radonić I. (2017). Population parameters and mito-nuclear mosaicism of *Anisakis* spp. in the Adriatic Sea. Mol. Biochem. Parasitol..

[14] Berland B. (1961). Nematodes from some norwegian marine fishes. Sarsia.

[15] Iglesias L., Valero A., Adroher F.J. (1997). Some factors which influence the in vitro maintenance of *Anisakis simplex* (Nematoda). Folia Parasitol..

[16] Iglesias L., Valero A., Benítez R., Adroher F.J. (2001). In vitro cultivation of *anisakis simplex*: Pepsin increases survival and moulting from fourth larval to adult stage. Parasitology.

[17] D’Amelio S., Mathiopoulos K., Santos C., Pugachev O., Webb S., Picanço M., Paggi L. (2000). Genetic markers in ribosomal DNA for the identification of members of the genus *Anisakis* (Nematoda: Ascaridoidea) defined by polymerase-chain-reaction-based restriction fragment length polymorphism. Int. J. Parasitol..

[18] Nadler S.A., D’Amelio S., Dailey M.D., Paggi L., Siu S., Sakanari J.A. (2005). Molecular phylogenetics and diagnosis of *Anisakis*, *Pseudoterranova*, and *Contracaecum* from northern Pacific marine mammals. J. Parasitol..

[19] Shamsi S., Gasser R., Beveridge I. (2013). Description and genetic characterisation of *Hysterothylacium* (Nematoda: Raphidascarididae) larvae parasitic in Australian marine fishes. Parasitol. Int..

[20] Anderson T.J.C. (2001). The dangers of using single locus markers in parasite epidemiology: *Ascaris* as a case study. Trends Parasitol..

[21] Gómez-Mateos M., Merino-Espinosa G., Corpas-López V., Valero-López A., Martín-Sánchez J. (2020). A multi-restriction fragment length polymorphism genotyping approach including the beta-tubulin gene as a new differential nuclear marker for the recognition of the cryptic species *Anisakis simplex* s.s. and *Anisakis pegreffii* and their hybridization event. Vet. Parasitol..

[22] Palomba M., Paoletti M., Webb S.C., Nascetti G., Mattiucci S. (2020). A novel nuclear marker and development of an ARMS-PCR assay targeting the metallopeptidase 10 (*nas 10*) locus to identify the species of the *Anisakis simplex* (s. l.) complex (Nematoda, Anisakidae). Parasite.

[23] Llorens C., Arcos S.C., Robertson L., Ramos R., Futami R., Soriano B., Ciordia S., Careche M., González-Muñoz M., Jiménez-Ruiz Y. (2018). Functional insights into the infective larval stage of *Anisakis simplex* s.s., *Anisakis pegreffii* and their hybrids based on gene expression patterns. BMC Genom..

[24] D’amelio S., Lombardo F., Pizzarelli A., Bellini I., Cavallero S. (2020). Advances in omic studies drive discoveries in the biology of anisakid nematodes. Genes.

[25] Romero M.C., Valero A., Navarro-Moll M.C., Martín-Sánchez J. (2013). Experimental comparison of pathogenic potential of two sibling species *Anisakis simplex* s.s. and *Anisakis pegreffii* in Wistar rat. Trop. Med. Int. Health.

[26] Arcos S.C., Ciordia S., Roberston L., Zapico I., Jiménez-Ruiz Y., Gonzalez-Muñoz M., Moneo I., Carballeda-Sangiao N., Rodriguez-Mahillo A., Albar J.P. (2014). Proteomic profiling and characterization of differential allergens in the nematodes *Anisakis simplex* sensu stricto and *A. pegreffii*. Proteomics.

[27] Detwiler J.T., Criscione C.D. (2010). An infectious topic in reticulate evolution: Introgression and hybridization in animal parasites. Genes.

[28] Kong Q., Fan L., Zhang J., Akao N., Dong K., Lou D., Ding J., Tong Q., Zheng B., Chen R. (2015). Molecular identification of *Anisakis* and *Hysterothylacium* larvae in marine fishes from the East China Sea and the Pacific coast of central Japan. Int. J. Food Microbiol..

[29] Farjallah S., Busi M., Mahjoub M.O., Slimane B.B., Paggi L., Said K., D’Amelio S. (2008). Molecular characterization of larval anisakid nematodes from marine fishes off the Moroccan and Mauritanian coasts. Parasitol. Int..

[30] Molina-Fernández D., Malagón D., Gómez-Mateos M., Benítez R., Martín-Sánchez J., Adroher F.J. (2015). Fishing area and fish size as risk factors of *Anisakis* infection in sardines (*Sardina pilchardus*) from Iberian waters, southwestern Europe. Int. J. Food Microbiol..

[31] MacKenzie K., Campbell N., Mattiucci S., Ramos P., Pinto A.L., Abaunza P. (2008). Parasites as biological tags for stock identification of Atlantic horse mackerel *Trachurus trachurus* L.. Fish. Res..

[32] Mattiucci S., Cimmaruta R., Cipriani P., Abaunza P., Bellisario B., Nascetti G. (2015). Integrating *Anisakis* spp. parasites data and host genetic structure in the frame of a holistic approach for stock identification of selected Mediterranean Sea fish species. Parasitology.

[33] Pekmezci G.Z., Onuk E.E., Bolukbas C.S., Yardimci B., Gurler A.T., Acici M., Umur S. (2014). Molecular identification of *Anisakis* species (Nematoda: Anisakidae) from marine fishes collected in Turkish waters. Vet. Parasitol..

[34] Costa A., Cammilleri G., Graci S., Buscemi M.D., Vazzana M., Principato D., Giangrosso G., Ferrantelli V. (2016). Survey on the presence of *A. simplex* s.s. and *A. pegreffii* hybrid forms in Central-Western Mediterranean Sea. Parasitol. Int..

[35] Cavallero S., Costa A., Caracappa S., Gambetta B., D’Amelio S. (2014). Putative hybrids between two *Anisakis* cryptic species: Molecular genotyping using High Resolution Melting. Exp. Parasitol..

[36] Bello E., Palomba M., Webb S.C., Paoletti M., Cipriani P., Nascetti G., Mattiucci S. (2021). Investigating the genetic structure of the parasites *Anisakis pegreffii* and *A. berlandi* (Nematoda: Anisakidae) in a sympatric area of the southern Pacific Ocean waters using a multilocus genotyping approach: First evidence of their interspecific hybridiza. Infect. Genet. Evol..

[37] Kim J.H., Kim J.O., Jeon C.H., Nam U.H., Subramaniyam S., Yoo S.I., Park J.H. (2018). Comparative transcriptome analyses of the third and fourth stage larvae of *Anisakis simplex* (Nematoda: Anisakidae). Mol. Biochem. Parasitol..

[38] Nam U.H., Kim J.O., Kim J.H. (2020). De novo transcriptome sequencing and analysis of *Anisakis pegreffii* (Nematoda: Anisakidae) third-stage and fourth stage larvae. J. Nematol..

